# Editorial: Beyond Reward: Insights from Love and Addiction

**DOI:** 10.3389/fpsyg.2016.01776

**Published:** 2016-11-15

**Authors:** Xiaochu Zhang, Zhiling Zou, Andreas J. Fallgatter

**Affiliations:** ^1^Chinese Academy of Sciences Key Laboratory of Brain Function and Disease, and School of Life Sciences, University of Science and Technology of ChinaHefei, China; ^2^School of Humanities and Social Science, University of Science and Technology of ChinaHefei, China; ^3^Center of Medical Physics and Technology, Hefei Institutes of Physical Science, Chinese Academy of SciencesHefei, China; ^4^Centers for Biomedical Engineering, University of Science and Technology of ChinaHefei, China; ^5^Faculty of Psychology, Southwest UniversityChongqing, China; ^6^Department of Psychiatry, University of TübingenTübingen, Germany; ^7^Graduate School LEAD, University of TübingenTübingen, Germany

**Keywords:** addiction, love, fMRI, EEG, reward system, sex

Rewarding stimuli promoting the learning of goal-directed behaviors tend to produce positive emotions, and subsequently repetition of those learned behaviors. Some kinds of drugs and behaviors are highly rewarding, and thereby, control human behavior by generating a state called addiction. The core feature of this state is compulsive behavior despite negative consequences. Addiction on a neurobiological level increases dopamine in the reward system and this is believed to underlie the rewarding effects. Large amount of studies in addiction have focused on the midbrain dopamine areas. Indeed, several researchers have defined addiction a disease of the reward system.

However, it has been argued that natural rewards can also induce an addictive-like state. For humans, natural rewards can be more complex than sex and food, and romantic love is interestingly proposed as a natural addiction. The following definition of romantic love as an addiction has been suggested: a positive addiction when one's love is reciprocated, non-toxic and appropriate, and a negative addiction when one's feelings of romantic love are socially inappropriate, toxic, not reciprocated, and/or formally rejected. Individuals in romantic love show many symptoms of drug and behavioral addictions, including tolerance, craving, emotional and physical dependence, relapse, and withdrawal. Human functional magnetic resonance imaging studies have shown that feelings of romantic love engage areas of the reward system, specifically dopamine-rich areas, including the midbrain, activated as well during drug and/or behavioral addiction.

It is an interesting topic to discuss addiction and love in the context of reward. In this e-book, we begin with an animal study of comparison between drug and natural reward. Duan et al. explore different effects of reward between morphine and food. A featured behavioral transition from psycho-activity to seeking behavior was shown during morphine abstinence, while only seeking behavior was displayed during food abstinence, suggesting that drug and natural rewards show some characteristics while mainly similarities exist. Lv et al. review cue reactivity in nicotine and alcohol addiction, suggesting that cultural cue reactivity may have an effect on addictive behavior through emotion and attention and is a field worth of exploring.

Some authors in this e-book seek to understand the reward system underlying behavioral addiction focusing on technology (mostly with internet addiction). Wang et al. find gray matter volume and white matter integrity altered in college students with mobile phone dependency. Li et al. present data showing that inhibitory control and reward functions, two associated cognitive processes, are impaired in problematic internet users, which strengthens the balance model of self-regulation theory. By using an addiction stroop task, Zhang et al. report that internet gaming disorder shows higher activations in brain areas involved in selective attention, visual processing, working memory, and cognitive control when facing internet gaming-related stimuli. Despite of local deficits, altered functional connectivity in internet addiction is also investigated. Lin et al. try to research the spontaneous brain activities of internet gaming disorder subjects and finds that these subjects show decreased functional connectivity in executive function and decision-making-related regions, which contributes to understanding the underlying pathophysiology. Treatment of internet addiction is an important area. Zhang et al. review studies on cue-induced behavioral and neural changes in internet gaming disorder, suggesting that mechanisms of internet gaming disorder mostly overlap with those of substance use disorder. The cue exposure therapy's effects in the treatment of addiction are also reviewed. Finally, an optimized paradigm for a probable treatment of internet gaming disorder is proposed.

The third part of this e-book addresses the topic of love. A review of addictive-like behaviors and brain systems associated with love is summarized by Fisher et al. A series of articles describe work aimed at understanding the neurobiology of love. Facial processing is closely related to romantic love. Wu et al. explore the effect of marriage style on the recognition of the beloved partner's face, especially in matriarchal societies. Marriage style affects the later stage processing of a beloved partner's face, which may be associated with greater affective arousal and familiarity. Another study by Sun et al. finds that facial attractiveness and expression are first processed in parallel for discrimination between stimuli. After the initial processing, more attentional resources are allocated to the faces with the most positive or most negative valence in both the attractiveness and expression dimensions. In the study by Song et al., early stage lovers show greater capacity for inhibiting action during presentation of negative emotional stimuli by comparing with individuals who are single, which may be related to the successful formation of romantic relationships.

Romantic love in a relationship is characterized by mate copying, attachment and intrasexual competition, which is investigated by several authors. Zhuang et al. confirm the mate copying effect in a behavioral experiment—greater increase in attractiveness ratings was observed for opposite-sex pictures in the interested than in the not-interested condition. And the fMRI results show that the DLPFC may be involved in the process related to mate copying. An electroencephalograph study by Hou et al. presents data suggesting that adult attachment styles affected individuals' recognition processing in response to love-related and sex-related images. Zheng et al. find that intrasexual competition can decrease pain empathic response to a same-sex “lucky guy” who has an attractive partner. Furthermore, right superior frontal gyrus and medial prefrontal cortex activations could predict participants' subsequent pain intensity ratings for the lucky guy.

Considered as a whole, the articles in this e-book demonstrate that romantic love may be considered a “natural addiction,” which parallels “diseases” of the reward system like drug and behavioral addictions in some respect. Drug and behavioral addictions are frequently related with negative consequences, while romantic love may be a positive addiction when the relationship is reciprocated, non-toxic and appropriate. This Research Topic brought together a range of perspectives regarding love and addiction. We hope love as a positive addiction offers a new view for future research in the field and that readers feel inspired by the articles in this e-book that provides a sample of such work.

## Author contributions

All authors listed, have made substantial, direct and intellectual contribution to the work, and approved it for publication.

### Conflict of interest statement

The authors declare that the research was conducted in the absence of any commercial or financial relationships that could be construed as a potential conflict of interest.

